# Nectar sugars and bird visitation define a floral niche for basidiomycetous yeast on the Canary Islands

**DOI:** 10.1186/s12898-015-0036-x

**Published:** 2015-02-01

**Authors:** Moritz Mittelbach, Andrey M Yurkov, Daniele Nocentini, Massimo Nepi, Maximilian Weigend, Dominik Begerow

**Affiliations:** Department of Geobotany, - LS Evolution & Biodiversity of Plants, Ruhr-University Bochum, ND 1/150 / Universitaetsstr. 150, 44780 Bochum, Germany; Leibniz Institute DSMZ - German Collection of Microorganisms and Cell Cultures, Braunschweig, Germany; Department of Life Sciences, University of Siena, Siena, Italy; Nees Institute for biodiversity of plants, Rheinische Friedrich-Wilhelms-Universität, Bonn, Germany

**Keywords:** Basidiomycetes, Bird pollination, Boraginaceae, Canary Islands, Nectar-dwelling yeast

## Abstract

**Background:**

Studies on the diversity of yeasts in floral nectar were first carried out in the late 19^th^ century. A narrow group of fermenting, osmophilous ascomycetes were regarded as exclusive specialists able to populate this unique and species poor environment. More recently, it became apparent that microorganisms might play an important role in the process of plant pollination. Despite the importance of these nectar dwelling yeasts, knowledge of the factors that drive their diversity and species composition is scarce.

**Results:**

In this study, we linked the frequencies of yeast species in floral nectars from various host plants on the Canary Islands to nectar traits and flower visitors. We estimated the structuring impact of pollination syndromes (nectar volume, sugar concentration and sugar composition) on yeast diversity.

The observed total yeast diversity was consistent with former studies, however, the present survey yielded additional basidiomycetous yeasts in unexpectedly high numbers. Our results show these basidiomycetes are significantly associated with ornithophilous flowers. Specialized ascomycetes inhabit sucrose-dominant nectars, but are surprisingly rare in nectar dominated by monosaccharides.

**Conclusions:**

There are two conclusions from this study: (i) a shift of floral visitors towards ornithophily alters the likelihood of yeast inoculation in flowers, and (ii) low concentrated hexose-dominant nectar promotes colonization of flowers by basidiomycetes. In the studied floral system, basidiomycete yeasts are acknowledged as regular members of nectar. This challenges the current understanding that nectar is an ecological niche solely occupied by ascomycetous yeasts.

**Electronic supplementary material:**

The online version of this article (doi:10.1186/s12898-015-0036-x) contains supplementary material, which is available to authorized users.

## Background

Several recent studies have invoked a resurgent interest in the importance of pollination to plant reproductive success and fertility [1,2]. Herrera et al. [3] and Vannette et al. [4] presented the first evidence for microbially-mediated impacts on plant pollination and fecundity by nectar dwelling yeast and bacteria, respectively. Nectar-dwelling unicellular fungi (yeasts) have fascinated researchers for over a hundred years. For example, ascomycetous yeasts, namely *Metschnikowia reukaufii* Pitt & M.W. Mill. and *Metschnikowia gruessii* Gim.-Jurado, were known since the late nineteenth century as common inhabitants of floral nectar in various host flowers [5]. Subsequent studies addressed the distribution [6], ecology [7-9], and physiological properties [10-12] of these species. Research in the past years added knowledge on functionality [13], population structure [14] and epigenetic variability of *Metschnikowia reukaufii* [15]. Studies on flowers all over the world strengthened the impression of a narrow and highly specific nectarivorous yeast community [16-18], which may consist up to 85% of fast growing ascomycetous specialists [19], adapted to sugar rich, temporally and spatially fragmented nectar environments.

A broader diversity of yeast species has been regularly reported from nectar [6,20,21], including both unicellular non-fermenting yeasts and yeast-like fungi [22,23]. Basidiomycetous yeasts are supposedly unable to persist in specific nectar environments based on their *in vitro* properties, such as growth preferences determined in culture media [24,25]. Thus, they are mostly regarded as autochthonous to non-flower habitats, such as plant surfaces or soils [23,26,27] and their presence in floral nectar is usually believed to be a contamination from other neighboring substrates [24].

The nectar of individual flowers represent species-poor habitats [18,19] characterized by both harsh physiological conditions [28] and strong species competition [29], which favors fast-growing microorganisms. The co-evolution of pollination syndromes between plants and flower visitors shaped a tremendous variety of floral habitats, which differ amongst other traits in nectar volume, and sugar concentration and composition [30,31]. Reukauf [7] and later Sandhu and Waraich [22] interpreted interspecific variation of nectar-traits as influential to nectar inhabiting fungi, however, Brysch-Herzberg [24] found no evidence for this in his exhaustive study. Since then, a few studies showed a correlation of main pollinator groups to yeast quantities [17,32], but not to the incidence of yeast nor their diversity [33].

The floral traits adapted to different pollination strategies should impose both direct and indirect effects on nectar dwellers. For example, Herrera et al. [28] showed the highly concentrated, sucrose-dominant nectar of *Helleborus foetidus* L. may act as a selective environmental filter for arriving yeast inoculum, which explains the dominance of osmophilic ascomycetes in nectar [16,34]. Responses of yeast community structure to nectar properties have received little attention so far, although different taxonomic groups of yeasts often possess distinct physiological characteristics [35] and assimilation tests of yeast strains demonstrated wide inter-specific differences in the ability to utilize various mono-, di, trisaccharides, as well as polyoles, alcohols etc. as a sole carbon source [36,37]. As a consequence, changes in the yeast environment, such as nectar chemistry, are likely to favor the growth of different yeast species, thereby directly affecting yeast community composition through the alteration of osmotic pressure, pH or availability of a particular nutrient.

The adaptation to environmental (nectar) habitats is only one factor responsible for a successful establishment of yeast populations. Equally important should be the propagation, the dispersal, and the inoculation of yeasts into nectar habitats, which are in turn indirectly driven by floral traits. Since nectar-dwelling yeasts are predominantly vectored by flower visitors [38], the composition and visitation frequencies of pollinator communities might conceivably govern the composition and abundances of the yeast-inoculum, respectively. Visitor anatomy and behavior should additionally impact yeast transfer and inoculation. Thus, a lower degree of visitor specialization signifies foraging on a wider variety of flowers or even food sources other than nectar. This might lead to a more heterogeneous pool of microorganisms, including ones normally not found in nectar, via the constant transfer of microorganisms between substrates [39].

To study the influence of pollination syndromes on nectar-dwelling yeasts, we analyzed floral nectars on the island of Tenerife, Spain. The Canary Islands provide a unique bird-pollination element [40], comprising opportunistic nectar feeding passerine birds [41]. Different evolutionary scenarios for the origin of ornithophily in Macaronesia have been proposed. Most likely, bird-related floral traits are relictual in some plant groups and de-novo in others (see [42] for Discussion). Flowers adapted to bird pollination are generally characterized by large red to orange corollas, diurnal anthesis, the absence of scent and the provision of suitable landing platforms [43]. Nectars are expected to be abundant, highly dilute with a dominance of monosaccharides [44]. However, on the Canary Islands, morphological adaptations of ornithophilous flowers are inconsistent with entomophilous relatives, albeit, Canarian bird species tend to prefer hexose (monosaccharide-dominated) to sucrose (disaccharide-dominated) nectars [45]. The de novo adaptation of ornithophily to passerine birds after island colonization is expected for the plant genus *Echium* L. (Boraginaceae) [46], which developed rather generalistic pollination syndromes and a variety of mixed bird/insect pollination systems [47].

In the present study we aim to link nectarivorous yeast diversity to different pollination-syndromes, addressing the impacts of nectar traits (volume, sugar concentration and composition) and floral visitors (frequency and composition). We hypothesize that yeast communities should be determined by two different sets of parameters: (i) alterations in the floral habitat itself, such as nectar concentration, abundance, and sugar composition, and (ii) the yeast transfer conditions as a result of different flower visitor assemblages.

## Results

### Yeast diversity

From 480 sampled flowers, 183 (38%) yielded culturable yeasts, resulting in 220 yeast isolates classified in 34 species (Additional file 1). A total of 13 (6%) identified yeasts were singletons (species found in a single sample only). Colonization frequencies differed considerably between years (16% in 2012 and 49% in 2013) and host plants: only 20% of *Isoplexis canariensis* (2013) flowers contained culturable yeast, while flowers of *Teucrium heterophyllum* (2013) yielded the highest percentage (75%). *Metschnikowia reukaufii* was the most frequent yeast species (n = 43), although only isolated in 2013. The survey yielded 32 strains of *Metschnikowia gruessii* (7 in 2012 and 25 in 2013) and 20 strains of *Cryptococcus carnescens* (8 in 2012 and 12 in 2013). The Shannon index of yeast diversity ranged from 1.0 in *Echium leucophaeum* (2013) to 2.4 in *Canarina canariensis* (2013) (Table 1). Most widespread yeast taxa were *Metschnikowia gruessii* and *Cryptococcus carnescens*, each isolated from flowers of six different host plants during the two years. *M. reukaufii* was found in five, while *Starmerella bombicola* and *Cryptococcus heimaeyensis* were found in nectars of four host plants. Mean yeast species richness in single flowers was 1.2 (±0.4 SD) with 3 species as the maximum richness per flower. Total yeast counts (CFUs per flower) ranged from 1 to approx. 1000 colonies per flower, but varied considerably within and between yeast species, flowers, and host plants (Additional file 1).

**Table 1:** **Sampled host plants, nectar traits, flower visitors, & diversity index**

		**Nectar traits**					**Visitation frequencies**					**Yeast diversity**
**Species**	**Year**	**Sucrose**	**Fructose**	**Glucose**	**Volume**	**Concentration**	**Bumblebees**	**Bees**	**Flies**	**Birds**	**InvSimpson**	**Shannon**
***Canarina***		*%*			*μL*	*%*	*visits/flower/minute*				*1/λ*	*H*
*C. canariensis*	2013	0.1	49.7	50.3	22.7	27.1	0.000000	0.000000	0.000000	0.003000	1.0	2.4
***Echium***												
*E. leucophaeum*	2012	8.7	45.5	42.7	4.5	14.5	0.000013	0.000258	0.000070	0.000077	2.3	1.7
*E. leucophaeum*	2013				6.3	17.2	0.000059	0.000257	0.000236	0.000000	2.4	1.0
*E. plantagineum*	2012	70.3	13.6	16.1	1.5	18.6	0.001384	0.000316	0.000109	0.000000	1.6	1.7
*E. plantagineum 1*	2013				2	19.2	0.000368	0.000247	0.000040	0.000000	2.2	1.3
*E. plantagineum 2*	2013				1.9	19.5	0.000346	0.000284	0.000072	0.000000	2.4	1.9
*E. simplex*	2013	4.4	45.7	49	8.8	14.6	0.000023	0.000222	0.000007	0.000107	2.1	1.9
*E. strictum*	2012	67.2	13.9	18.3	1.2	24.9	0.000900	0.002742	0.000135	0.000000	1.7	1.7
*E. strictum*	2013				0.9	24.8	0.002863	0.000800	0.000139	0.000000	1.6	1.9
***Isoplexis***												
*I. canariensis*	2013	0.1	42.9	55.7	5.4	31.1	0.000000	0.000000	0.000000	0.011000	1.0	1.6
***Lavatera***												
*L. acerifolia*	2013	0.1	48.7	51.3	3.5	42.7	NA	NA	NA	NA	NA	1.2
***Teucrium***												
*T. heterophyllum*	2013	78.9	10.7	10.4	3.5	42.7	NA	NA	NA	NA	NA	1.1

### Nectar analysis

Analysis of main nectar-sugars (sucrose, glucose, and fructose) revealed two major groups of host plants with either sucrose-dominant or hexose-dominant nectars (Table 1). Nectar-volumes ranged from 0.9 μl (±0.2 SD) in *E. strictum* to 22.7 μl (±17) in *C. canariensis* with sugar concentrations from 14.5% (±2.6) in *E. leucophaeum* to 42.7% (±10.4) in *T. heterophyllum*.

### Observations of floral visitors

We observed a total of 7503 flower visits on the 4 focal *Echium* host species. Individual visitation rates differed between the observed species up to one magnitude (Table 1). *Echium strictum* received the highest visitation frequency in 2012 (0.00378 visits per flower per minute (v/f/min) ±0.052 SD) and *Echium simplex* the lowest in 2013 (0.00037 ± 0.004). Most abundant pollinator groups were bumblebees (0.0056 ± 0.001), consisting almost exclusively of visits by *Bombus canariensis* Pérez. Visitors of the functional group of bees (0.00485 ± 0.001) were classified as members of the genera *Megachile* and *Osmia*, while honeybees were only present in *Echium strictum* in 2012 and account for only 21% of all bee visits. Flower visiting birds were identified either as Common Chiffchaff (*Phylloscopus collybita* Vieillot), Atlantic Canary (*Serinus canaria* L.), or Blue Tit (*Parus caeruleus* L.; only on *Echium leucophaeum*) and have been observed on *Echium leucophaeum* in 2012 (0.0001 ± 0.003) and on *Echium simplex* in 2013 (0.00011 ± 0.003).

### Yeast communities and pollination syndromes

**Figure 1:**
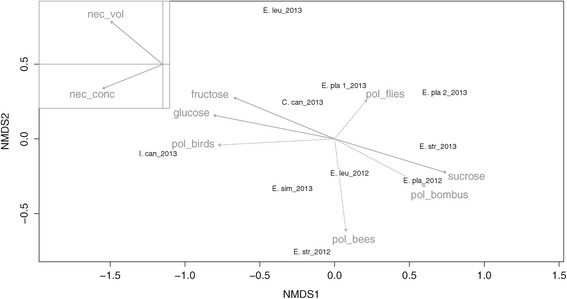
**Ordination plot of yeast diversity.** First and second axis of non-parametric multidimensional scaling of yeast diversity based on a Bray-Curtis dissimilarity matrix (stress: 0.09). The plot shows respective host-plants and sampling years. Arrows correspond to fitted nectar traits (solid lines) and flower-visitor frequencies (dotted lines): arrow directions are gradients and lengths are proportional to the correlations between variables and the ordination. Nectar volume and sugar concentration are shown separately without distortion for the sake of lucidity.

**Figure 2:**
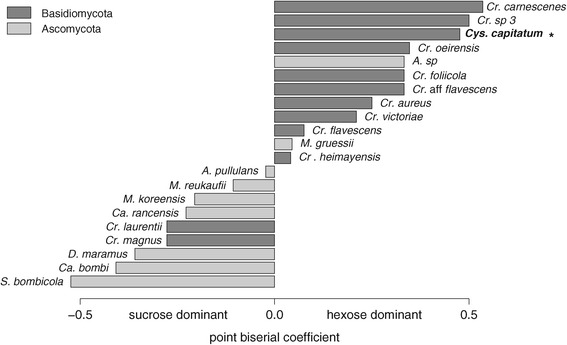
**Point biseral coefficient plot.** Point-biserial correlation coefficients of ascomycetous and basidiomycetous yeast species calculated for nectar type (hexose- or sucrose-dominant) of isolation substrate. Significant correlations of distribution and the respective factor level is indicated by * *p* < 0.05.

**Figure 3:**
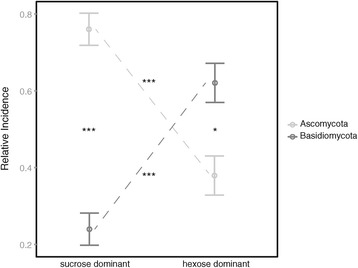
**Relative incidences of orders.** Relative incidence of ascomycetous and basidiomycetous yeast species in all sampled host plants, plotted according to respective nectar-type. Bars are standard deviations and middle points the respective mean. Significant differences (Wilcoxon pairwise tests and subsequent Holm correction) are indicated by * *p < 0.05*, and *** *p < 0.001*).

Nectar traits and visitation frequencies, in our study defined as pollination syndromes are correlated (Mantel test: *r = 0.426; p < 0.01*). This correlation impedes separate analyses of their impacts on nectar dwelling yeast communities, although the ordination plot (Figure 1) suggests that yeast species frequencies are clearly structured by sampled nectar traits (axis 1) and flower visitors (axis 2). Consequently, frequencies of yeast isolation in our study are significantly driven by nectar sugar type (*PERMANOVA*: *R*^*2*^ = *0.179*, *p < 0.05*) although only *Cys. capitatum* seems to be significantly related to hexose dominant nectars (Figure 2). The nectar type also significantly discriminates the relative incidences of ascomycetous versus basidiomycetous yeasts in our study (Figure 3).

**Figure 4:**
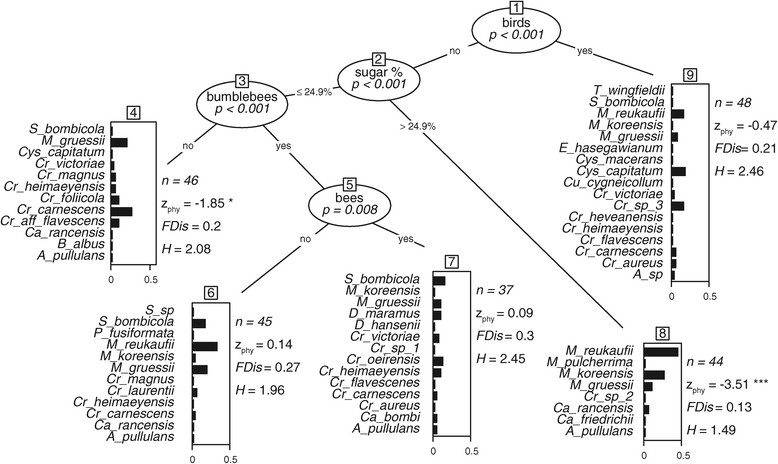
**Partitioning tree of yeast diversity.** Recursive binary partitioning tree calculated for yeast diversity predicted by nectar traits (volume + sugar concentration + nectar type), pollinator compositions (visitation frequencies of each functional group), and year. Stopping criteria were set to α = 0.01 and minimum sizes of terminal nodes restricted to 30 L. Nodes are numbered and respective critical splitting variable values are provided on the lines. Bar-plots show weights of species in the respective node assemblages. Light bars illustrate basidiomycetes and dark bars ascomycetes. Number of isolates (n), Functional dispersion (FDis), Shannon index (H), and mean pairwise distances compared to patterns expected under the selected null model (Z_phy_) are provided for each species assemblage. Asteriks refer to significanes (* *p* < 0.05, *** *p* < 0.001) as calculated to the null model with default iterations and randomizations.

In addition to the nectar sugar concentration, frequencies of visitors to flowers (birds, bees, bumblebees) influence yeast diversity (Figure 4). Although specialized nectar dwelling members of the Metschnikowiaceae lineage are ubiquitous, they form a phylogenetically clustered node assemblage in flowers with high sugar concentrations and are responsible for a reduced functional yeast diversity (FDis) in this substrate. The occurrence of these yeasts varied substantially between sampling years, with most pronounced fluctuation being observed for *M. reukaufii*. Nonetheless, this species was isolated from a total of 27 samples of host plants, which were not part of the analysis in 2012.

## Discussion

The overall diversity of yeast species determined in this study is consistent with results of previous studies [19,48]. However, our study yielded numerous basidiomycetous yeasts regularly isolated from flowers and identified as *Cryptococcus carnescens*, *Cr. heimaeyensis*, and *Cystofilobasidium capitatum* (Additional file 1). Our results show that yeast communities are significantly mediated by the type of flower visitor and by nectar sugar concentration (Figure 4). This confirmed former hypotheses of flower-trait mediated yeast communities [7,22] and expanded the known effects of pollinator composition [17] and pollination syndromes [32] on the diversity and composition of yeast communities. Although floral traits and pollinator composition are naturally correlated, they may steer two different mechanisms of the yeast-colonization process, namely (i) the ability to grow in nectar and (ii) the probability of flower inoculation. Below, we discuss the two mechanisms in more details.

**Figure 5:**
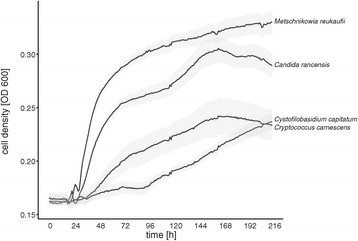
**Growth curves of selected strains.** Growth curves of selected strains in artificial nectar medium with 40% sugar concentration measured as OD 600. Shaded areas correspond to standard errors calculated of 16 replicates.

(i) Among other natural yeast harboring substrates, such as plant surfaces, fruits, and soils [26,27,49], nectar habitats stand out by high sugar concentrations, microaerophilic conditions, low nitrogen levels, and the widespread presence of anti-microbial compounds [50]. As has been shown by Peay et al. [29], these environmental conditions may regulate the growth of nectar dwelling microorganisms, giving nectar a filtering property for inoculated colonizers. Our results supported this hypothesis, as increased sugar concentrations favored the growth of only a few highly specialized ascomycetes (Figure 4). We also isolated strains of *Cr. carnescens* and *Cys. capitatum* from sugar rich nectars (Additional file 1) and showed positive growth in kinetic laboratory experiments with up to 40% sugar, which indicated their ability to grow in nectars of all studied host plants (Figure 5). Together with our results that basidiomycetous yeasts are significantly more successful in colonizing hexose dominant nectars (Figure 3), we conclude that sugar concentration alone cannot explain the composition of yeast communities in floral nectars. Additional factors, such as sugar composition (Figure 2) and a prolonged flower lifetime might be important selective filters of yeast communities in the sampled ornithophilous host plants. The latter should favor the establishment of basidiomycetes in nectar, accounting for their slower growth and extended lag phase in kinetic growth experiments in comparison to tested ascomycetous specialists (Figure 5).

(ii) Selective effects of nectar can only partially explain the reduced occurrence of ascomycetes in hexose-dominant nectars. Specialized nectar yeasts are believed to grow equally well in different nectar-sugar compositions ([24], Figure 2), although data on host-genotype interaction of *M. reukaufii* provides some support to the diversifying selection hypothesis [51] and suggests growth characteristics to be rather strain-specific.

The dominance of basidiomycetes in hexose nectars might be a result of an altered flower visitor community (Figure 4) since the visitation frequency of insects, commonly vectoring and inoculating ascomycetes [24,28] is reduced. In addition, the inoculation probability of allochthonous species should be increased in ornithophilous flowers. This might be caused by the generalistic foraging behavior of nectar-feeding birds on the Canary Islands, which feed on a broad variety of resources of plant origin, such as fruits or plant tissues [52] in addition to hexose-rich nectar [45]. Since these plant-related habitats harbor large numbers of basidiomycetous yeasts [26,27], the probability of yeast inoculation in nectar is increased by bird visitors. Indeed, South African plants, visited by passerine birds were found to harbor more yeasts (incidence and abundance) than sympatric plants visited by insects, only [32]. Yeast diversity in our study is either high in flowers visited by birds or in flowers visited by bumblebees and other bees (Figure 4). Our observations mirror one common ecological law that selective pressure in the environment constrains species diversity, including microorganisms. Less strict conditions (sugar type and concentration) attract different flower visitors and allow a broader range of microbes to colonize flowers from a larger number of sources.

The shift from insect to passerine bird pollination on the Canary Islands resulted in various degrees of dependence to bird-visits, ranging from strict ornithophily in *Isoplexis canariensis* [42] to occasional visits by birds in *Echium wildpretii* [53]. As a consequence, floral adaptations to ornithophilous pollinators might be imperfect in the sense of classic pollination ecology: for example *Teucrium heterophyllum* is believed to be pollinated by passerine birds, despite highly concentrated sucrose-dominant nectar. Taken together, these diverse and overlapping floral habitats provide a broad spectrum of available vectors and niches for microbial nectar-colonizers on a small regional scale. Our study reveals that the filter effect of nectar [28] might depend on nectar properties and on the diversity of the microbial inoculate. The pollination syndromes of the sampled host plants could in turn facilitate the inoculation and ease establishment of allochthonous microorganisms in nectars due to their species richness and overlapping diversity. These suggestions are supported by increased functional diversities of yeast communities in niches other than high-concentrated nectars (Figure 4).

Despite being combined in one ornithophilous pollination-syndrome, passerine bird pollination in the old world and hummingbird pollination in the new world evoked different floral adaptations by plants, impeding comparisons of the diversity of nectar-dwelling microbes. Sugar-concentrations of nectars in hummingbird-flowers have been found to be elevated (25%) in contrasts to sunbird pollinated flowers (21%) [54]. In addition, hummingbirds commonly prefer sucrose-dominated nectars [44,55] and forage on flowers with long and narrow corollas, which impede the visitation of other floral visitor-groups [56].

Indeed, nectars of hummingbird-pollinated *Mimulus aurantiacus* are dominated by specialized ascomycetes, such as *M. reukaufii* and *Candida rancensis* [18], species prevailing in sucrose-dominated flowers in our study (Figure 2). Nonetheless, Belisle *et al.* [57] showed that hummingbirds transport a large diversity of microfungi, including yeasts species isolated in our survey, namely *C. rancensis*, *S. bombicola*, *Cr. flavescens*, *Cr. carnescens*, and *A. pullulans* (Additional file 1).

### Basidiomycetes in nectar

**Figure 6:**
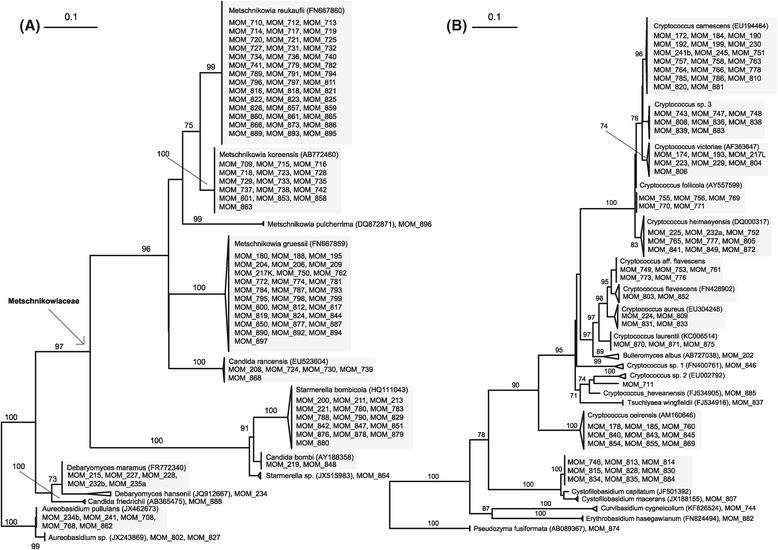
**Phylogenetic trees of isolated strains.** Maximum-likelihood trees of LSU (D1/D2 domains) sequences of isolated strains and their closest NCBI-BLAST hits of **(A)** ascomycetes and **(B)** basidiomycetes. Shaded areas illustrate species delimitation used in the study, when more than one strain identified. The numbers given on branches are frequencies (>70%) with which a given branch appeared in 1000 bootstrap replications. The scale indicates the number of expected substitutions accumulated per site.

Several studies acknowledge only ascomycetes from the order Metschnikowiaceae and phylogenetically related species of the genus *Candida* as specialized nectar-dwelling yeasts [24,25,28]. The high number of isolates per species in this group underlines the expected specialization (Figure 6). The repeated isolation of a broader diversity of yeast and yeast-like species from flowers contrasts this view and suggests that additional groups of organisms might have exploited the vast number of different floral microhabitats evolved within the multitude of pollination syndromes in Angiosperms [22,58]. This hypothesis is supported by the high frequencies and within-flower abundances of ‘allochthonous’ species in this study (Additional file 1). Total cell densities of almost all isolated yeast species, measured as CFUs per flower show a considerable inter- and intraspecific variability, as has also been reported from other plants [18,58,59]. Interestingly, high yeast colony numbers were not restricted to fermenting ascomycetes in our study, but were also common for several basidiomycetous species in ornithophilous flowers (Additional file 1).

According to our results, *Cys. capitatum* and *Cr. carnescens* are common inhabitants of sampled flowers and may directly profit from the sampled hexose-dominant nectar-environments (Figure 2). Brysch-Herzberg [24] also isolated *Cys. capitatum* from floral nectar regularly, without recognizing its potential nectar-related habit. Previously there was no evidence that *Cys. capitatum* might be competitive with fast-growing, fermenting ascomycetes in sugar-rich environments, due to characteristics of its former isolation habitats, such as soil [60,61] and marine surface water [62]. However, recently, researchers have documented an affinity to sugar-enriched habitats by *Cys. capitatum* and report this species from fruits of *Sorbus aucuparia* L. and *Rosa canina* L. [63,64], tree exudates ([65] and references therein), and fruiting bodies of the tree parasite *Cyttaria* [66].

Little is known about the ecology of the second frequent basidiomycete, *Cr. carnescens,* which was isolated from flowers twice [57,67], and has been reported as frequent inhabitant of grapes [68] and phyllosphere in Mediterranean ecosystems [69]. Despite these plant related sources, *Cr. carnescens* has been understood as a pervasive species isolated from seawater, soil, and glacial ice [69]. Based on its phenotype, this yeast has been long considered a synonym of *Cr. laurentii* until Takashima et al. [70] demonstrated that this complex comprises several distinct and distantly related species. Although proper interpretation of both *Cr. laurentii* and *Cr. carnescens* from older studies is therefore precluded, several members of the two phylogenetic clades (*Cr. laurentii* and *Cr. victoriae*, respectively) inhabit substrates of plant origins, such as fruits and leaves [69] and were also isolated from nectar in the present study: *Cr. laurentii*, *Cr. tephrensis*, *Cr. foliicola*, and *Cr. heimaeyensis*. The latter species was also reported from flowers before [24] but mislabeled as *Cryptococcus* aff. *victoriae* (Additional file 2).

## Conclusion

In this study, we present significant trends in the community structure of nectar dwelling yeast. Nectar sugar concentration, composition, and flower visitor assemblages were identified as main selective forces. Furthermore, we reveal the previously underestimated importance of basidiomycetous yeasts as inhabitants of ornithophilous flowers with hexose dominant nectar on Tenerife. Bird-pollination on the Canary Islands clearly represents an exotic case study in the evolution of floral traits, but the provision of hexose-rich or even dominant nectar is a common phenomenon and can be found in various plants [44], e.g. related to dipteran pollination syndromes [71] or due to phylogenetic history [72]. More comprehensive data on yeast distribution across different pollination syndromes and nectar types would be clearly desirable to better comprehend the distribution, ecology, diversity and functions of basidiomycetous yeasts in floral nectar.

It is widely known that basidiomycetous and ascomycetous yeasts differ substantially in their lifestyles and physiological properties, suggesting different ecological strategies. While basidiomycetes have been hardly associated with nectar foraging insects, ascomycetous specialists have been almost exclusively isolated from flowers, honey pots and insects [24,73]. This leads to the conclusion that ascomycetes spend their whole life cycle inside the insect-flower system, whereas basidiomycetes might possess a broader variety of alternative substrates or even switch from saprobic to parasitic or fungicolous lifestyles [74,75]. Nonetheless, both groups highly depend on durable structures to overcome phases of transportation and rest in ephemeral nectar habitats. While the formation of ascospores in ascomycetous yeasts has been well studied, similar resistant structures of basidiomycetes in sugar-rich habitats have not been identified so far. Whether or not these ecological prerequisites together with the corresponding assimilation profiles provide basidiomycetes an advantage in colonizing nectars of ornithophilous plants requires detailed studies.

Inconsistencies in yeast incidences among years, the unbalanced experimental design, and the reliance on data from literature in this study clearly formulate the need for a more detailed and comprehensive sampling. Nonetheless, diversity patterns of nectar-borne yeasts remain stable during both years, validating our conclusions although impeding broader generalization.

### Experimental procedures

#### Study sites & plant species

Fieldwork was conducted on the island of Tenerife in the eastern Anagar mountain region. In April 2012 a sympatric population of *Echium strictum* L.f., *E. leucophaeum* Webb ex Sprague & Hutch., and *E. plantagineum* L. was studied close to Chinamada (approx. 28°33.80, − 016°17.41'). In May 2013, we sampled a sympatric population (approx. 28°34.70', −016°08.75') of *E. strictum*, *E. leucophaeum*, *E. simplex* DC., and *E. plantagineum* (1 population in scrubland and 1 population close to forest). All other studied taxa were sampled within 500 m of the focal population. Plant species grow in natural sclerophyllous coastal scrubland, except for *Isoplexis canariensis* (L.) Lindl. and *Canarina canariensis* (L.) Vatke, which are part of the vegetation of lower laurel forests [76]. A complete list of sampled plants can be found in Additional file 1.‬‬‬‬

#### Yeast isolation

Individual flowers (flowering branches or inflorescences) in fertile female stage were carefully removed (to avoid mechanical damages) from 3 plant individuals (except for *C. canariensis* (n = 6) and *E. plantagineum* (n = 4)) in the late afternoon and immediately covered in sterile plastic-bags until further processing in the lab. To account for biases due to different flower numbers of host plants, we randomly picked 40 flowers from collected plant material for nectar sampling. In adition, we covered 5 flowers of each host plant in bud stage and processed the nectar as controls. Nectar was removed from the flowers using sterile micro capillaries (Hirschmann Laborgeräte, Eberstadt, Germany) within a maximum of 4 hours after flower harvest. Total nectar volume of each flower was mixed with in 100 μl of sterile tap water and streaked out on modified solid YM medium (0.3% w/v Yeast extract, 0.5% w/v Peptone, 0.3% w/v Malt extract, 1% w/v Glucose, 2% w/v Agar) supplemented additionally with nectar-related sugars (1% w/v Fructose and 1% w/v Sucrose) and acidified with 1% v/v 80% Lactic acid (final pH = 4.5) to prevent bacterial growth. Plates were stored at room temperature for 4 days and then kept at lower temperature (4°C) to slow down the development of molds. Plates were examined after 7 days of incubation: colonies were differentiated into macro-morphological types using dissection microscopy and the respective counts were recorded as colony forming units (CFU). One representative per plate was transferred into pure culture. All isolated strains were stored at −80°C in glycerol/glucose (1:1, w:w). Nectar samples from covered buds (controls) did not yield any fungal or yeast cultures.

#### Yeast identification

Pure cultures were transferred to liquid YM-medium and incubated for 48 hours at room temperature. DNA was extracted using a phenol-chloroform extraction method and the LSU ribosomal gene region (D1/D2 domains) was amplified (for detailed methods see [77]) and sequenced using the primers ITS1f or NL1 and NL4 [78,79] .

Sequences were edited manually and trimmed using Sequencher 5.0 following the criteria: (i) trimming no more than 25% of the sequence length until the first 25 nucleotides would contain less than 5 ambiguities, and (ii) trimming no more than 25% of the sequence length until the first 25 nucleotides would contain less than 5 nucleotides with confidences below 25. Two separate alignments for Basidiomycota and Ascomycota were created using MAFFT 7.110 [80], manually edited and curated with GBlocks allowing smaller final blocks and gap positions within it [81]. For convenience, formal classification into operational taxonomic units (OTU) was conducted using MOTHUR 1.32.1 [82] applying a 98% cut-off value and considering also different similarity values traditionally used to delimit yeast species in ascomycetes and basidiomycetes [18,83,84]. Results of the OTU analysis were confirmed by morphological inspections and interpretation of phylogenetic maximum likelihood trees obtained with raxmlGUI [85] using the GTRGAMMA model and 1000 bootstrap replications (Figure 6).

Three strains with reduced sequence qualities (MOM_217, MOM_232, MOM_859) were manually inspected again with the Sequencher 5.0 software and included into an alignment of closely related OTUs taking into account both their morphological characterization and phylogenetic placement. Representative sequences for each OTU were identified to the species level using NCBI GenBank and MycoBank databases [86]. Sequences are stored at the EMBL nucleotide sequence database [87] and representative strains are deposited in the DSMZ, German Collection of Microorganisms and Cell Cultures (Braunschweig, Germany) (Table 1).

#### Nectar analysis

Standing crop nectar was removed from 25 randomly selected flowers per species and population, harvested at the same time and from the same individual plants as before. Volumes were measured with glass-capillary tubes. Sugar concentrations were estimated with a handheld refractometer (10-80% brix, neoLab Universal, Germany). To analyze sugar compositions, 5 flowers were covered with nylon meshes in bud stage to avoid nectar contamination. Nectar samples were harvested from open flowers and stored in sterile tubes, filled with 70% Ethanol until further analysis by HPLC (see [71] for detailed methods). Nectar samples for sugar analysis from *Echium leucophaeum*, *E. simplex*, *E. strictum* were collected at the Botanical Garden in Berlin-Dahlem, Germany and samples of *E. plantagineum* at the Botanical Garden of Bonn-University, Germany, following the same procedure. Nectar sugar compositions of *Canarina canariensis*, *Teucrium heterophyllum* L’Hér., *Lavatea acerifolia* Cav., and *Isoplexis canariensis* are taken from literature [45,47].

#### Observations of flower visitors

Observations of floral visitors were conducted prior to nectar yeast samplings on the same individual plants. Each flower visit was counted as a new and independent event without any regard to individual visitors probing on more than one flower per plant in a row. Flower observations were undertaken in 10 min intervals with 3 researchers simultaneously, each one observing a different plant species. Focal species and individuals were changed every 30 minutes to ensure an objective threshold and to provide coverage of all plant species during all times of one day. Pollinators were pooled to functional groups as proposed by Fenster *et al.* [31], since we believe this classification is suitable for the objectives of this study. To increase accuracy, large plants were divided into intercepts to reduce the number of flowers observed simultaneously. Observations on *Canarina canariensis* and *Isolplexis canariensis* did not yield visitor observations, data regarding visitation rates of these species was therefore taken from Ollerton *et al.* [41] and Rodríguez-Rodríguez & Valido [42], respectively. *Teucrium heterophyllum* is reported as generally bird-pollinated [88,89], and we rely on this information since no observational records were available for this plant species. Similarly, *Lavetera acerifolia* is considered insect-pollinated [90].

#### Growth tests

Growth tests were conducted in closed 96-well microplates [91] in 150 μl of artificial nectar medium, consisting of yeast nitrogen base (YNB, Difco BD) and 40% sugars mixture (Glucose, Sucrose, Fructose, 1:1:1 w/w) using Infinite 200 Pro microplate reader (Tecan Austria GmbH, Austria). Cells from 5-day cultures were harvested from solid YM media dissolved in 1% PBS buffer, filtered through 30 μm filter (Partec GmbH, Germany) and inoculated in the artificial nectar medium (20 cells per μL = 3000 cells per well) using BD FACSAria III cell sorter (BD Biosciences, USA) as starting cultures. Each strain was inoculated in 16 wells and a total of 32 wells were blank containing the medium only. Cultures were incubated for 10 days at 25°C and measured automatically every hour. Between measurements plates were incubated as static culture for 45 minutes followed by 15 minutes shaking at 1000 rpm with 4 mm amplitude prior to the next absorbance measurement. Absorbance was measured at 600 nm every hour using the following options: multiple per well (12 reads in circle (filled) pattern) and 5 flashes in a read. Values from the reads were averaged.

#### Data analysis

The incidence of species was determined in all 480 flowers and organized in a presence/absence ‘site*species’ matrix to analyze yeast diversity. A total of 291 flowers did not yield any culturable yeast and were excluded from the analysis. To avoid biased results in the final analysis due to inflated zero counts and unequal sample sizes, yeast incidences of single flowers of each host plant and year were summarized and handled as yeast frequencies per host plant and year. Relative incidences were determined as a proportion of a particular yeast species frequency in each host plant and year. We calculated Shannon’s index of diversity [92] to characterize the diversity and structure of host-specific yeast communities.

Mean nectar volume and sugar concentration for each host plant and year were standardized to account for differences in measured units. Except for nonparametric multidimensional scaling (nmds) analysis, the sugar composition was classified based on the percentages of sucrose and hexose into a factor variable (nectar type), providing 4 categories reaching from sucrose dominant to hexose-dominant nectar (for details, see [55]).

Pollinator observations of individual plants were pooled to achieve ‘visitation frequencies’ of each functional visitor group as visits/flower/minute (v/f/min) for each host plant and year. To assess diversity of flower visitors for each host plant, we calculated the generalization level according to [93] as Simpson diversity index.

Mantel tests of dissimilarity matrices of yeast species frequencies (Bray-Curtis), as implemented in the R package ‘vegan’ [94] were used to evaluate the correlation between floral traits and flower-visitor frequencies. To create an ordination plot of yeast diversity, we applied the ‘metaMDS’ function for nonparametric multidimensional scaling (nmds) to the dissimilarity matrix. Floral traits and visitor frequencies were subsequently added to the nmds graphic using the implemented ‘envfit’ function. The function ‘adonis’ was used to partition the variation of yeast frequencies among factorized floral traits and visitation frequencies. To account for differences between samplings in 2012 and 2013, we constrained the subsequent permutation tests (10,000 replicates) to the respective sampling years. Both functions are also implemented in R package ‘vegan’.

We used recursive binary partitioning to regress yeast diversity according to environmental covariates incorporating nectar traits (volume + sugar concentration + nectar type), pollinator compositions (visitation frequencies of each functional group), and sampling year. The procedure constructs unified tests for independence by means of conditional distribution of linear statistics in the permutation test framework. Stopping criteria were set to the nominal level of the conditional independence tests as α = 0.01, using a simple Bonferroni correction. The analysis was conducted with the ‘ctree’ function implemented in the ‘party’ package [95]. Subsequently, we extracted assembled yeast communities from the recursive partitioning analysis output and used the function ‘ses.mpd’, as implemented in the R package ‘picante’ [96] to test whether these assemblages represent phylogenetically clustered subsamples. The ‘ses.mpd’ calculates the mean pairwise distance between all species in the subsamples, based on a provided phylogenetic maximum likelihood tree, and compares phylogenetic relatedness to patterns expected under a null model, allowing for randomized community data matrix abundances within samples (maintaining species occurrence frequencies). Additionally, we calculated the functional dispersion indices for all node assemblages (‘FDis’ in the ‘FD’ package [97]) of assimilation traits for each acknowledged species.

Differences in relative incidence between ascomycetes and basidiomycetes were calculated with the Kruskal Wallis Test, using nectar type as grouping factor. Significances for single factor combinations were identified by Wilcoxon pairwise tests with subsequent Holm correction for multiple tests [98].

We calculated the point-biserial correlation coefficient to analyze the species ecological preferences [99]. The index measures the association of standardized species distributions for site groups as implemented in the r-package ‘indicspecies’ [100]. All calculations were accomplished using the R framework 3.0.2. [101].

### Ethics statement

All research was conducted within an appropriate ethical framework. Field work on Tenerife was conducted with permission by the Área de Medio Ambiente, Sostenibilidad Territorial y Aguas (Expte: AFF 33/13, No Sigma: 2013–00172).

### Availability of supporting data

The data set supporting the results of this article is available in the Data Dryad repository, doi:10.5061/dryad.0qp3q [102].

## Additional files

Additional file 1:
**Yeast diversity.** Frequencies of isolated ascomycetous and basidiomycetous species determined for each host plant and year are plotted in the upper line of each cell. The lower line shows mean CFUs per host plant and year and ranges in brackets. The total number of isolates per host species (column) or yeast species (line) is labeled with “n”. Nucleotide sequence accession numbers, and DSMZ collection numbers are provided for each representative strain, as given by the OTU analysis. Additional file 2:
**Identification of putative new yeast species.** Yeast cultures were identified using nucleotide sequences of the D1/D2 domains of the large subunit (26S/28S or LSU). For species identification, the nucleotide sequences were compared with sequences deposited in the NCBI and MycoID databases, respectively. Alignments were made using the MAFFT algorithm [80]. Phylogenetic analyses were performed with MEGA 6.06 software [103]. Substitution model (Kimura two-parameter, K2 + G) was derived from model test implemented in this software. Missing data was partially (95%) deleted. Phylogenetic placement was supported by 100 rounds of bootstrap replicates. The closest match (similarity, number of substitutions and gaps) among publically available sequences is derived from the pair-wise comparison of the two closest nucleotide sequences using blastn (NCBI) suite.
