# Supplementary material for: Nectar sugars and bird visitation define a floral niche for basidiomycetous yeast on the Canary Islands
Source: BMC Ecol. 2015 Feb 1;15(1):2. doi: 10.1186/s12898-015-0036-x (PMC4318194; doi:10.1186/s12898-015-0036-x)
Supplement: Additional file 2: — Identification of putative new yeast species. Yeast cultures were identified using nucleotide sequences of the D1/D2 domains of the large subunit (26S/28S or LSU). For species identification, the nucleotide sequences were compared with sequences deposited in the NCBI and MycoID databases, respectively. Alignments were made using the MAFFT algorithm [80]. Phylogenetic analyses were performed with MEGA 6.06 software [103]. Substitution model (Kimura two-parameter, K2 + G) was derived from model test implemented in this software. Missing data was partially (95%) deleted. Phylogenetic placement was supported by 100 rounds of bootstrap replicates. The closest match (similarity, number of substitutions and gaps) among publically available sequences is derived from the pair-wise comparison of the two closest nucleotide sequences using blastn (NCBI) suite. [file 12898_2015_36_MOESM2_ESM.docx]

**Additional File 2: Phylogenetic placements of undescribed *Cryptococcus* species**

**(i) *Cryptococcus* sp. 1**


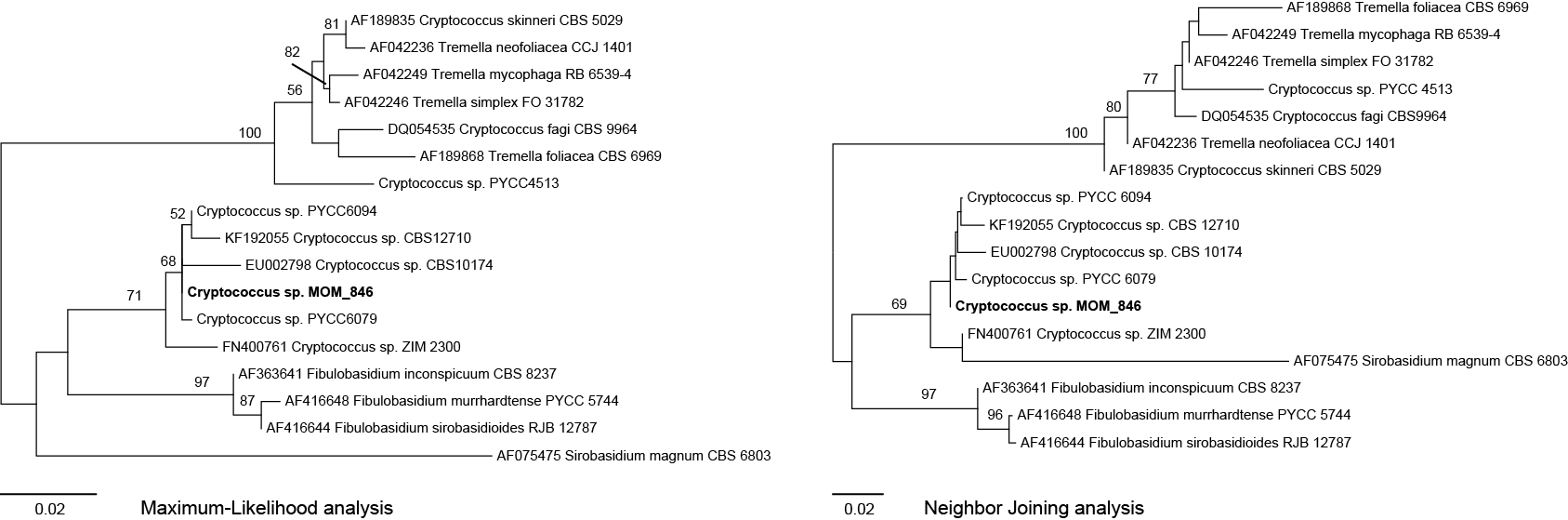


Isolate: MOM_846

GenBank: HG421439

Strain: DSM 27418

Phylogenetic placement: Sirobasidium-Fibulobasidium clade, Tremellales, Tremellomycetes, Agaricomycotina, Basidiomycota

Closest match: *Cryptococcus* sp. PYCC 6079 (http://pycc.bio-aware.com) with 99% similarity, including 2 nucleotides substitutions and 1 gap

**(ii) *Cryptococcus* sp. 2**


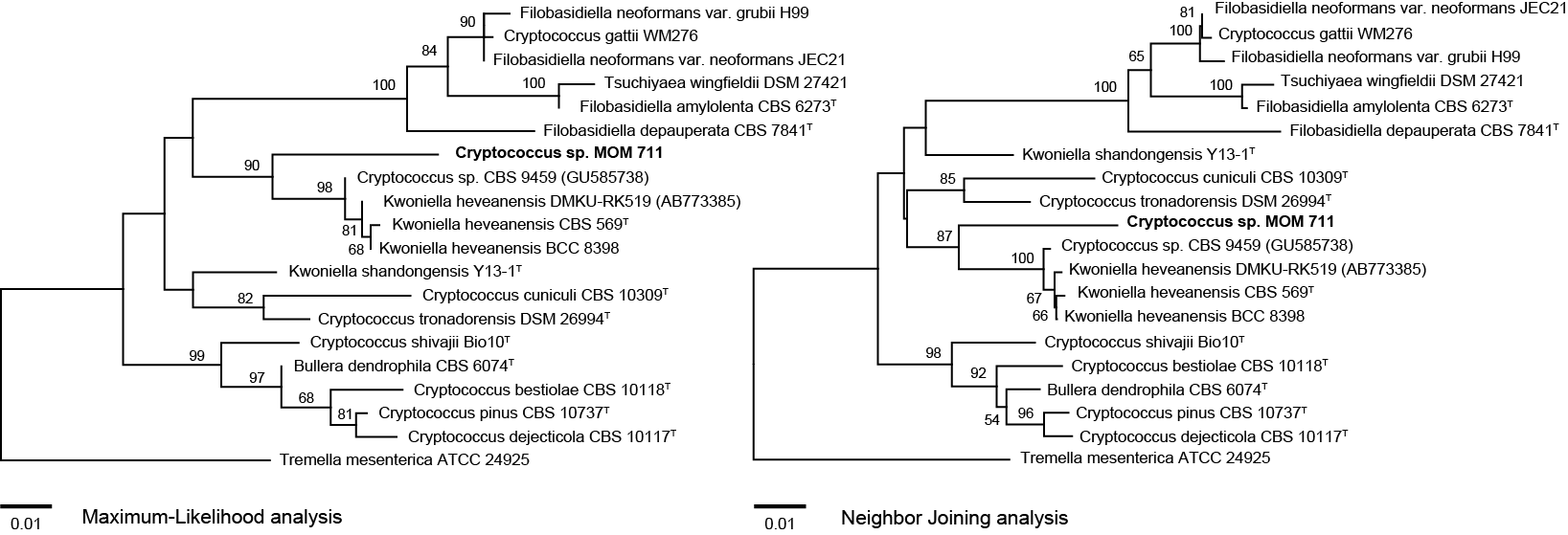


Isolate: MOM_711

GenBank: HG421441

Strain: DSM 27419

Phylogenetic placement: Kwoniella-Filobasidiella clade, Tremellales, Tremellomycetes, Agaricomycotina, Basidiomycota

Closest match: *Cryptococcus* sp. CBS 9459 (GU585738) with 95% similarity, including 26 nucleotides substitutions and 2 gaps

**(iii) *Cryptococcus* sp. 3**


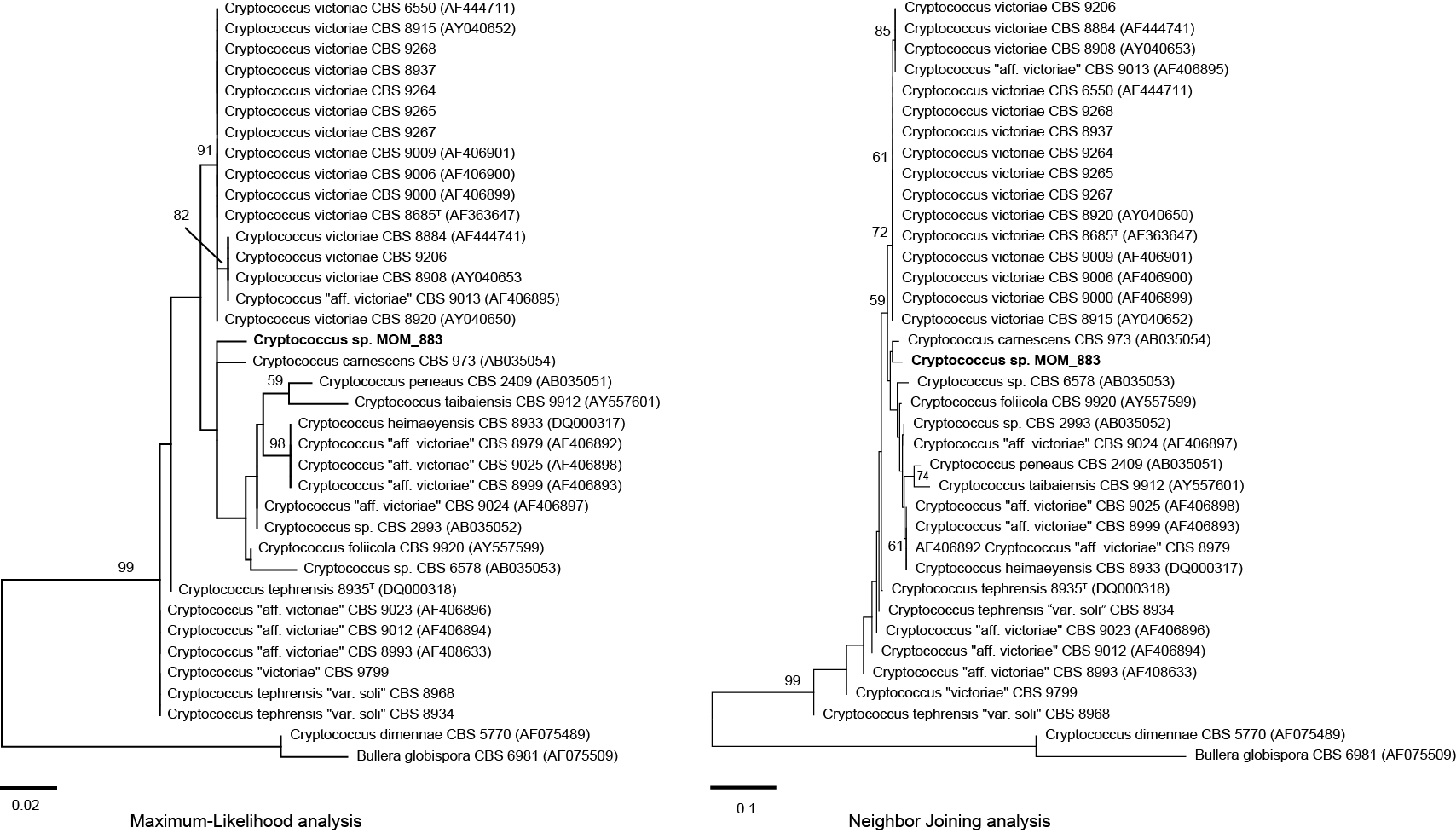


Isolate: MOM_883

GenBank: KM408428

Strain: DSM 29302

Phylogenetic placement: Victoriae clade, Tremellales, Tremellomycetes, Agaricomycotina, Basidiomycota (Text S2, Figure 1)

Closest match: *Cryptococcus carnescens* CBS 973 (AB035054) with 98% similarity, including 10 nucleotides substitutions

Further isolates in this study: MOM_743, MOM_747, MOM_748, MOM_808, MOM_836, MOM_838, MOM_839
